# The relationship mammalian p38 with human health and its homolog Hog1 in response to environmental stresses in *Saccharomyces cerevisiae*

**DOI:** 10.3389/fcell.2025.1522294

**Published:** 2025-03-10

**Authors:** Gang Du, Kaifang Zheng, Cunying Sun, Mingyue Sun, Jie Pan, Dan Meng, Wenqiang Guan, Hui Zhao

**Affiliations:** Tianjin Key Laboratory of Food Biotechnology, College of Biotechnology and Food Science, Tianjin University of Commerce, Tianjin, China

**Keywords:** HOG pathway, human health, osmotic stress, MAPK, p38

## Abstract

The mammalian p38 MAPK pathway plays a vital role in transducing extracellular environmental stresses into numerous intracellular biological processes. The p38 MAPK have been linked to a variety of cellular processes including inflammation, cell cycle, apoptosis, development and tumorigenesis in specific cell types. The p38 MAPK pathway has been implicated in the development of many human diseases and become a target for treatment of cancer. Although MAPK p38 pathway has been extensively studied, many questions still await clarification. More comprehensive understanding of the MAPK p38 pathway will provide new possibilities for the treatment of human diseases. Hog1 in *S. cerevisiae* is the conserved homolog of p38 in mammalian cells and the HOG MAPK signaling pathway in *S. cerevisiae* has been extensively studied. The deep understanding of HOG MAPK signaling pathway will help provide clues for clarifying the p38 signaling pathway, thereby furthering our understanding of the relationship between p38 and disease. In this review, we elaborate the functions of p38 and the relationship between p38 and human disease. while also analyzing how Hog1 regulates cellular processes in response to environmental stresses. 1, p38 in response to various stresses in mammalian cells.2, The functions of mammalian p38 in human health.3, Hog1 as conserved homolog of p38 in response to environmental stresses in *Saccharomyces cerevisiae*. 1, p38 in response to various stresses in mammalian cells. 2, The functions of mammalian p38 in human health. 3, Hog1 as conserved homolog of p38 in response to environmental stresses in *S. cerevisiae*.

## Introduction

Cells have evolved sophisticated sensory mechanisms and information transduction systems to respond to environmental challenges and ensure survival (Nadal and Posas, 2015). Eukaryotic cells, ranging from yeast to mammals, the multiple mitogen-activated protein kinase (MAPK) cascades play a crucial role in regulating various cellular processes (de Nadal et al., 2002; Westfall et al., 2004).

**FIGURE 1:**
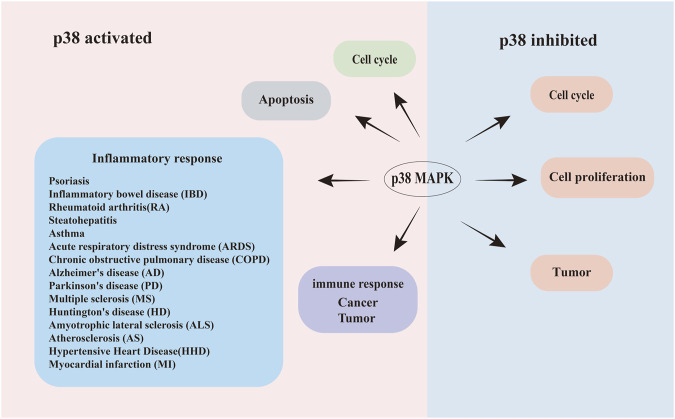
Schematic illustration of the bidirectional roles of p38 signaling in human diseases. Dual regulatory roles of p38 MAPK activation and inhibition in disease pathogenesis. Left panel (Activation): phosphorylated p38 (Red area) drives pathological processes, including: apoptosis dysregulation, acceleration or retardation of the cell cycle, Inflammatory disorders (Psoriasis, inflammatory bowel disease (IBD), rheumatoid arthritis (RA), steatohepatitis, asthma, acute respiratory distress syndrome (ARDS), and chronic obstructive pulmonary disease (COPD). Neuroinflammatory/neurodegenerative diseases: Alzheimer’s disease (AD), Parkinson’s disease (PD), multiple sclerosis (MS), Huntington’s disease (HD), amyotrophic lateral sclerosis (ALS), atherosclerosis (AS), hypertensive heart disease (HHD), and myocardial infarction (MI)), tumor progression, and cancer cell proliferation. Right panel (Inhibition): pharmacological suppression of p38 (blue area) attenuates disease progression through: enhanced cellular survival, slowed tumor growth. Arrows indicate directional signaling flow.

In mammals, p38 MAPK is one of the most significant signaling pathways in the MAPK cascade (Obata et al., 2000; Chen et al., 2001; Kyriakis and Avruch, 2001; Pearson et al., 2001; Johnson and Lapadat, 2002; Goldsmith et al., 2004). p38 MAPK is involved in multiple essential functions including apoptosis, cytokine production, transcriptional regulation, and cytoskeletal reorganization (Figure 1). This pathway also regulates the activity and expression of key inflammatory mediators, including cytokines and proteases, which are critical for cancer progression (Sheikh-Hamad and Gustin, 2004; Obata et al., 2000) as well as diseases related to inflammatory responses (Wang et al., 2024), including colitis, arthritis, atherosclerosis, lung diseases, human immunodeficiency virus infection, Alzheimer’s disease (AD) (Hugon and Paquet, 2021), and cell carcinoma (Martínez-Limón et al., 2020), tumors (Bulavin and Fornace, 2004) among other diseases (Figure 1). Givern its critical role in these processes, p38 MAPK has been extensively studied as a therapeutic target, with p38 inhibitors explored for clinical treaments (Han et al., 2020; Asih et al., 2020).

Interestingly, the p38 MAPK pathway has a homologous counterpart in *Saccharomyces cerevisiae*, where the high osmolarity glycerol (HOG) pathway performs a similar function (Galcheva-Gargova et al., 1994; Cooper, 1994; Han et al., 1994; de Nadal et al., 2011). The HOG pathway, a yeast-specific MAPK signaling cascade, is crucial for the cell’s response to environmental stressors such as osmotic stress (de Nadal et al., 2011). The key protein in this pathway, Hog1, is a conserved homologue of p38 (Han et al., 1994). The similarities between the mammalian p38 MAPK pathway and the yeast HOG pathway provide valuable insights into the conserved nature of MAPK signaling across species, and understanding these pathways in yeast may offer new directions for studying p38 MAPK in human diseases. This discussion will first explore the p38 pathway and its relationship to human diseases, while also examining how the HOG MAPK pathway responds to various external stimuli, providing new insights and directions for studies on p38. How MAP kinase p38 affects human health.

### p38 and cellular processes

p38 was originally identified as a protein with a molecular weight of 38 kDa, characterized by the rapid phosphorylation of its tyrosine residues in response to various environmental stimuli (Han et al., 1994). These stimuli include heat shock, changes in osmotic pressure, oxidative stress, genotoxic agents and DNA-damaging agents such as cisplatin, adriamycin, ultraviolet light, and *γ*-radiation (Kyriakis and Avruch, 2012). Additionally, p38 is activated by inflammatory cytokines, pathogen-associated molecular patterns (PAMPs), danger-associated molecular patterns (DAMPs) (Martínez-Limón et al., 2020) and lipopolysaccharide (LPS) stimulation (Han et al., 1994). p38 MAPK represents a group of highly conserved protein kinases, and phosphorylated p38 MAPK can activate a diverse range of substrates, including transcription factors, protein kinases, and various cytoplasmic and nuclear proteins (Obata et al., 2000; Coulthard et al., 2009).

**FIGURE 2:**
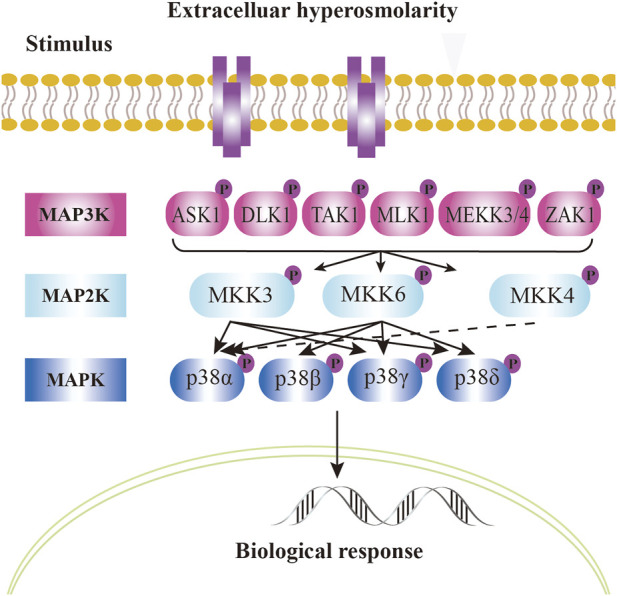
p38 signal pathway in mammalian cells. Mammalian p38 kinases consist of four proline - directed serine/threonine kinases, namely, p38*α*, p38*β*, p38*γ*, and p38*δ*, which are encoded by four genes. The classical activation of p38 follows a three - tiered mechanism. The stimuli for p38 activation are cellular stressors, such as oxidative stress, inflammatory stimuli/cytokines, ultraviolet radiation, and cell membrane osmotic pressure. Upstream stimuli activate MAP kinases (MAPKKK), such as kinases like ASK1, TAK1, MLK, etc. These kinases, in turn, phosphorylate and activate MAPKK, such as MKK3, MKK4, or MKK6. MKK, in turn, phosphorylates and activates p38 kinases on threonine and tyrosine residues in the activation loop. In the case of p38*α*, it is activated by MKK4 (dashed arrow). The dual phosphorylation of p38 can be detected by phosphorylation - specific antibodies and serves as a marker of p38 activation. Dual - phosphorylated p38 is fully active and targets downstream phosphorylation substrates, altering their structure, activity, function, localization, or interaction with other biomolecules, thus regulating cellular responses. The phosphorylated state is represented by “P”. Solid arrows and dashed arrows indicate the directionality of the signal.

In mammalian cells, four homologous p38 MAPK proteins are encoded by different genes: p38*α*. (MAPK14), p38*β* (MAPK11), p38*γ* (MAPK12), and p38*δ* (MAPK13) (Cuenda and Rousseau, 2007) (Figure 2). These proteins are broadly expressed, yet their expression patterns differ across tissues. p38*α* is universally expressed in all cell types, while p38*β* is predominantly found in the brain, thymus, and spleen. p38*γ* is abundant in skeletal muscle, and p38*δ* levels are higher in the pancreas, intestines, adrenal glands, kidney and heart (Mertens et al., 1996; Goedert et al., 1997; Jiang et al., 1997; Beardmore et al., 2005; Cuenda and Rousseau, 2007; Cuenda and Sanz-Ezquerro, 2017). The external environment activates MAP3K, including TAK1 (Moriguchi et al., 1996), ASK1 (Ichijo et al., 1997), DLK (Hirai et al., 1997), MLK3 and ZAK1 (Doza et al., 1995; Brancho et al., 2003). This activation leads to the stimulation activation of upstream MAP2K, specifically MKK3 and MKK6, which in turn activates the downstream p38 kinase, resulting in their phosphorylation (Figure 2). All four p38 kinases possess conserved Thr-Gly-Tyr (TGY) biphosphorylated motifs (Han et al., 2020). Notably, among of these four types of p38 MAPK, p38*β* is exclusively phosphorylated by MKK6, while only p38*α* is specifically activated by MKK4 (Enslen et al., 1998; Alonso et al., 2000; Cuadrado and Nebreda, 2010) (Figure 2).

Activated p38 rapidly accumulates and translocates to the nucleus, where it phosphorylates various transcriptional regulators that coordinate specific gene expression programs (Ono and Han, 2000; Cuenda and Rousseau, 2007). Additionally, it may interact with other signaling pathways, binding to non-p38-regulated transcription factors to trigger diverse responses, including inflammation, cell cycle arrest, apoptosis, senescence, cytokine production and RNA splicing regulation (Coulthard et al., 2009; Sanz-Ezquerro and Cuenda, 2021) (Figure 1). Notably, strong and sustained p38 activation is linked to apoptosis, senescence and terminal cell differentiation (Puri et al., 2000; Haq et al., 2002). In contrast, low-level p38 activation supports cell survival (Puri et al., 2000; Haq et al., 2002; Macé et al., 2005).

### Cell cycle influenced by p38 MAPK

When cells are exposed to stress, defects in cell growth occur as cell cycle checkpoint systems and protective responses are activated. Energy is redirected from other cell functions to support the stress response. In mammalian cells, the p38 MAPK pathway plays a pivotal role in regulating cell cycle progression under various stress conditions, such as osmotic stress, reactive oxygen species, DNA damage and aging. These stresses lead to defects in cell cycle delay, impairing cell viability. (Duch et al., 2012; Barnum and O'Connell, 2014; Martínez- Limón et al., 2020). p38 MAPK is particularly important for regulating cell proliferation during the G1/S and G2/M phases of the cell cycle. (Barnum and O'Connell, 2014; Martínez- Limón et al., 2020). For instance, p38 MAPK enhances cell survival by downregulating the expression of the retinoblastoma (RB) tumor suppressor gene, which is a key regulator of G1 phase restriction point in metastasis (Ambrosino and Nebreda, 2001; Gubern et al., 2016).

In the G1/S transition, p38 interacts with several key regulatory proteins, such as Cyclin D1, Cdc25A, and p53, to modulate cell cycle progression. (Bulavin and Fornace, 2004). Cyclin D1, encoded by the human CCND1 gene, is essential for the G1/S transition (Tchakarska and Sola, 2020). Inhibition of p38 MAPK during this transition lead to a downregulation of cyclin D1 levels, slowing the conversion from G1 to S phase, thereby impeding cancer cell cycle progression and reducing cancer incidence (Lavoie et al., 1996). Additionally, p38 MAPK regulates the stability of Cdc25A, its activation can inhibit Cdc25A activity, further slowing cell cycle proliferation (Galaktionov et al., 1995; Mailand et al., 2000; Bartek and Lukas, 2001; Zhao et al., 2002; Goloudina et al., 2003). Notably, Cdc25A is often overexpressed in primary human breast cancer, where it could serve as a potential therapeutic target (Cangi et al., 2000). Meanwhile, p53 functions as a tumor suppressor at the G1/S checkpoint by upregulating proteins such as p21Cip1/WAF1, GADD45, and 14-3-3*σ*, which prevents progression to S phase and induce G1 phase stagnation (Takekawa et al., 2000; Ho and Benchimol, 2003; Stramucci et al., 2018).

Furthermore, the G2/M checkpoints are crucial for halting mitotic progression in response to chromatin damaged or incomplete replication (Bulavin and Fornace, 2004). Studies indicate that p38*α* is involved in G2/M checkpoints, promoting cell cycle arrest and facilitating DNA repair (Wagner and Nebreda, 2009). Experiments with transgenic mice expressing an active *mkk6*Δ mutant in immature thymus cells have demonstrated sustained activation of the p38 MAPK pathway inhibits both cell cycle progression and differentiation (Diehl et al., 2000; Ambrosino and Nebreda, 2001). Despite these findings, the precise molecular mechanisms through which p38 MAPK integrates with other key cell cycle checkpoints, including the DNA damage response and tumor suppressor signaling pathways, remain incompletely understood. Further research is needed to elucidate these interactions and their implications for cell cycle regulation under stress conditions.

### Apoptosis influenced by p38 MAPK

Apoptosis is a genetically controlled, multistep process of cell death that eliminates damaged cells while preventing inflammatory response (Obata et al., 2000). In many biological systems, the activation of p38 MAPK activity is associated with apoptosis, whereas its inhibition can reduce apoptosis events (Ziegler-Heitbrock et al., 1992; Cardone et al., 1997; Obata et al., 2000). The role of p38 MAPK in apoptosis may depend on the mode and duration of its activation (Obata et al., 2000). For instance, brief activation of p38 MAPK promotes erythroid differentiation of SKT6 cells, while prolonged activation leads to apoptosis (Nagata and Todokoro, 1999; Macé et al., 2005). Similarly, early activation of p38 MAPK can prevent apoptosis in neutrophils treated with tumor necrosis factor (TNF)-*α*, whereas later activation appears to facilitate apoptosis in these cells (Roulston et al., 1998).

Some studies suggest that p38 MAPK influences apoptosis both upstream and downstream of cysteine protease (Cardone et al., 1997; Ziegler-Heitbrock et al., 1992), which are central to the apoptosis pathway and exist as inactive zymogens (Fernandes-Alnemri et al., 1996; Cahill et al., 1996). Moreover, activation of MEKK can stimulate the p38 signaling pathway, promoting apoptosis in T cells and fibroblasts (Huang et al., 1997). Interestingly, heat stress has been shown to inhibit LPS-induced apoptosis by blocking the calpain/p38 MPAK pathway (Liu et al., 2016). Overall, the p38 MAPK pathway plays a crucial role in regulating cell fate, yet the precise mechanism underlying this process remains to be fully elucidated.

## p38 in diseases

### Inflammatory response regulated by p38 MAPK

The p38 MAPK pathway is recognized for its complex role in the inflammatory response, where it is involved not only in promoting inflammation but also in mediating anti-inflammatory processes. Activation of p38 MAPK can stimulate the expression of transcription factors such as AP-1 (Garcia et al., 1998; Yang et al., 2014) through pro-inflammatory mediators, including interleukin-1 (IL-1), IL-6, TNF. This stimulation further enhances the production of pro-inflammatory cytokines, thereby intensifying the inflammatory response (Guzman-Martinez et al., 2019; Chan et al., 2020; Liao et al., 2021). Concurrently, p38 MAPK also plays a role in regulating anti-inflammatory mediators such as IL-10 and transforming growth factor-*β* (TGF-*β*), which may inhibit inflammation under certain conditions (Guzman-Martinez et al., 2019; Chan et al., 2020; Liao et al., 2021). Due to this dual functionality, p38 MAPK is considered a key regulator of the inflammatory response (Garcia et al., 1998; Yang et al., 2014). The NLRP3 inflammasome is a multiple protein complex that detects pathogens and danger signals, promoting the maturation and release of inflammatory factors such as IL-1*β* (Ising et al., 2019). Abnormal activation of the NLRP3 inflammasome has been linked to various inflammatory diseases, including AD (Ising et al., 2019). The p38 MAPK signaling pathway exhibits a dual role in the activation and expression of the NLRP3 inflammasome (Wang et al., 2024). Specifically, phosphorylation of p38 MAPK enhances the function of the NLRP3 inflammasome, leading to a heightened inflammatory response (Wang et al., 2024). Conversely, the inhibition or absence of p38 MAPK may result in excessive activation of the NLRP3 inflammasome during its activation phase, which can exert an anti-inflammatory effect through the regulation of mitochondrial Ca^2+^ uptake (Chanjitwiriya et al., 2020).

Additionally, the p38 MAPK signaling pathway plays a crucial role in the functional activation, proliferation and migration of macrophages, as well as in regulating their phagocytic capabilities, which are essential for inflammatory responses (Senokuchi et al., 2005; Hou et al., 2022; Wang et al., 2024). Increased phosphorylation levels of p38 MAPK enhance both the phagocytic ability of macrophages and their production IL-10. Specifically, p38*α* is vital in mediating inflammatory responses, notably in conditions such as psoriasis, while p38*β* has also been implicated in various inflammatory diseases, (Johansen et al., 2005), including inflammatory bowel disease (IBD), rheumatoid arthritis (RA), steatohepatitis (Zhang and Reynolds, 2019; Otsuka et al., 2010; Liang et al., 2013), asthma, acute respiratory distress syndrome (ARDS) and chronic obstructive pulmonary disease (COPD). Furthermore, studies have highlighted the complex pro-inflammatory and anti-inflammatory roles of p38*γ* and p38*δ* in cytokine production during innate immune responses (Escós et al., 2016), particularly in collagen-induced arthritis (CIA) (Han et al., 2020). These isoforms regulate the expression of cytokines, chemokines, and inducible nitric oxide synthases, which are crucial for the innate inflammatory response against infections by controlling the expression of multiple protein-coding genes involved in the activation, recruitment of immune cells, and the elimination of pathogens in bone marrow derived macrophages (Risco et al., 2012; Alsina-Beauchamp et al., 2018).

Inhibiting p38 MAPK can help prevent inflammation and the death of muscle fiber, thus providing a potential treatment for various forms of muscular dystrophy forms of muscular dystrophy (Brennan et al., 2021). Microglia, the innate immune effector cells of the central nervous system, undergo phenotypic changes and release inflammatory mediators, which are pivotal in neuroinflammation associated with conditions such as stroke (Park et al., 2015). The p38 MAPK pathway is integral to the functioning of microglia and other cell types (Park et al., 2015; Kheiri et al., 2018; Yan and Zhao et al., 2020; Yang et al., 2020; Fan et al., 2021; Gaikwad et al., 2021; Li et al., 2020; Lo et al., 2022). Neuroinflammation significantly contributes to neurodegenerative diseases, including AD (Peel et al., 2004), Parkinson’s disease (PD) (Thomas et al., 2008), and multiple sclerosis (MS), with chronic oxidative stress further exacerbating neurodegenerative changes (Solleiro-Villavicencio et al., 2018). Aditionally, p38 MAPK signaling is implicated in the pathogenesis of other neurodegenerative diseases, such as Huntington’s disease (HD) and amyotrophic lateral sclerosis (ALS) (Perregaux et al., 1995; Johnson and Bailey, 2003; Hollenbach et al., 2004; Culbert et al., 2006; Van Eldik et al., 2007; Thomas et al., 2008; Feng et al., 2019), as well as various cardiovascular diseases, including atherosclerosis (AS), obesity-related cardiac hypertrophy (ORCH) (Wang et al., 2016), myocardial infarction (MI) and cerebrovascular disease (Bassi et al., 2008; Muslin, 2008). Moreover, p38 MAPK is particularly important in the context of chronic pain diseases (Coulthard et al., 2009).

### The role of MAP kinase p38 in regulation of immune response

The importance of the p38 signaling pathway in regulating the immune response has garnered significant attention in the context of carcinogenesis (Martínez-Limón et al., 2020). Immune cells can profoundly influence tumor progression through the secretion of cytokines and chemokines (Martínez-Limón et al., 2020).

The effects of p38 on tumor are complex and multifaceted.

First, the activation of p38 can promote tumorigenesis. For instance, p38*α* regulates the induction of the pro-inflammatory mediator Cyclooxygenase-2 (COX-2), which may contribute to cancer progression in various cancers, including non-melanoma skin cancer, breast cancer, and glioma (Bachelor and Bowden, 2004; Timoshenko et al., 2006; Xu and Shu, 2007). Additionally, p38*α* can inhibit inflammation-related intestinal epithelial damage and tumorigenesis, while also promoting the proliferation and survival of colon cancer cells (Bachstetter et al., 2011). Furthermore, p38*γ* expression is crucial for proliferation of colon cancer (Chen et al., 2000) and liver tumors (Tang et al., 2005). Inhibition of p38 has been shown to decrease the expression of TGF-*β*-dependent MMP- 9, thereby reducing bone metastasis of breast cancer in mouse models (Suarez-Cuervo et al., 2004), and preventing bone metastasis of prostate cancer cells (Arechederra et al., 2015). Chronic inflammatory diseases, particularly those affecting the gastrointestinal tract, are associated with an increased risk of cancer development (Grivennikov and Karin, 2011). The p38 pathway regulates the production of key cytokines such as TNF, IL-6, IL-1, COX-2, IL-17 and other cytokines, which play significant roles in tumor growth, survival, and tumorigenesis (Martínez-Limón et al., 2020).

Second, the activation of p38 can inhibit tumor occurrence. The p38*α* and p38*β* subtypes (Ambrosino and Nebreda, 2001) inhibit G0, G1/S and G2/M cell cycle checkpoint control, leading to growth arrest and induction of apoptosis (Bulavin and Fornace, 2004; Kummer et al., 1997; She et al., 2001) or senescence (Wang et al., 2002; Haq et al., 2002; Bulavin et al., 2002). p38 downregulates the expression of cyclin through phosphorylation, thereby inhibiting cell proliferation across various cancer cell lines (Gubern et al., 2016). Moreover, p38*α* can limit the proliferation of hematopoietic stem cells (Tamura et al., 2000), cardiomyocytes (Engel et al., 2005) and pancreatic islets (Wong et al., 2009). Additionally, constitutive activation of the p38 MAPK pathway, through MKK3 or MKK6, can induce senescence in several cell types (Wang et al., 2002; Haq et al., 2002) and inhibit tumor formation.

Thus, p38 not only has the capacity to inhibit tumor cell proliferation, but also act as a tumor promoter (Martínez-Limón et al., 2020). Experimental evidence suggests that low p38 activity in the early stages of cancer may facilitate tumor formation and growth, while increased activation of this pathway in advanced tumor stages may be beneficial (Igea and Nebreda, 2015).

## Therapeutic targets of p38

Due to its crucial role in regulating cellular functions, p38 is currently being extensively studied as a drug target, and various inhibitors (Genovese, 2009) are being investigated for the treatment of diseases such as pain, asthma, cognitive impairment, RA, PD (Coulthard et al., 2009), cancer, myelodysplastic syndrome and depression (Johansen et al., 2005). In 2011, the European Commission approved Esbriet (pirfenidone), identified as a p38*γ* inhibitor, for the treatment of idiopathic pulmonary fibrosis (Moran, 2011). Another notable example is Ralimetinib (or LY2228820), a potent and selective inhibitor of p38*α* and p38*β,* utilized as either a single agent or in combination therapy for ovarian cancer, glioblastoma and metastatic breast cancer (Vergote et al., 2020). Furthermore, p38*α* inhibitors may be beneficial in treating tumors reliant on the progression of p38 MAPK activity, potentially enhancing the efficacy of DNA-damaging chemotherapy by inhibiting p38α-mediated cell cycle arrest and affecting DNA repair mechanisms (Wagner and Nebreda, 2009).

Despite these advancements, our understanding of the p38 signaling pathway remains limited. Nonetheless, novel drug targets for p38 kinase or its downstream components continue to be promising candidates for the development of new therapies addressing a wide range of human diseases.

## HOG MAPK

Many signaling pathways present in yeast have equivalent systems in mammalian cells, exhibiting extensive functional conservation (Han et al., 1994; Sheikh-Hamad and Gustin, 2004; Jiménez et al., 2020). Mammalian p38 MAPK is both structurally and functionally homologous of yeast HOG MAPK (Galcheva-Gargova et al., 1994; Han et al., 1994; Nadal and Posas, 2015). Notably, it has been reported that p38 can complement the normal functions of HOG MAPK in mutant yeast strains (Han et al., 1994). Consequently, in depth studies of the HOG signaling pathway in yeast, which serves as the conserved homologue of p38 in mammalian cells, may provide novel insights for disease treatment targeting p38.

In *S. cerevisiae*, the HOG signaling pathway has been extensively investigated. It coordinates multiple cellular functions, regulates cell survival and growth, and plays a crucial role in osmotic signaling (Nadal and Posas, 2022). Initially, the HOG signaling pathway in *S. cerevisiae* was thought to be activated solely by osmotic stress. However, recent studies have revealed that Hog1 is also activated by various other environmental stresses, including heat (Winkler et al., 2002; Yamamoto et al., 2008; Dunayevich et al., 2018), cold (Hayashi and Maeda, 2006; Panadero et al., 2006), oxidative (Singh, 2000; Haghnazari and Heyer, 2004; Lee et al., 2017), acid (citric acid (Lawrence et al., 2004), acetic acid (Mollapour and Piper, 2006)), heavy metals [e.g., copper (Ren et al., 2022), cadmium (Jiang et al., 2014; Zhao et al., 2021), iron (Martins et al., 2018)], metalloid (arsenic (Sotelo and Rodríguez-Gabriel, 2006; García et al., 2004; Lee and Levin, 2018), antimony (Thorsen et al., 2006)), LPS (Marques et al., 2006), curcumin (CUR) (Azad et al., 2014), hypoxia (Hickman et al., 2011) and caffeine (Elhasi and Blomberg, 2023), methylglyoxal (MG) (Aguilera et al., 2005), KP1019 (Singh et al., 2014) and carbon stress (Vallejo and Mayinger, 2015), cesium chloride (Del Vescovo et al., 2008). In this section, we primarily describe the HOG signaling pathway and its activation by various environmental stresses.

### HOG-MAPK signaling pathway

**FIGURE 3:**
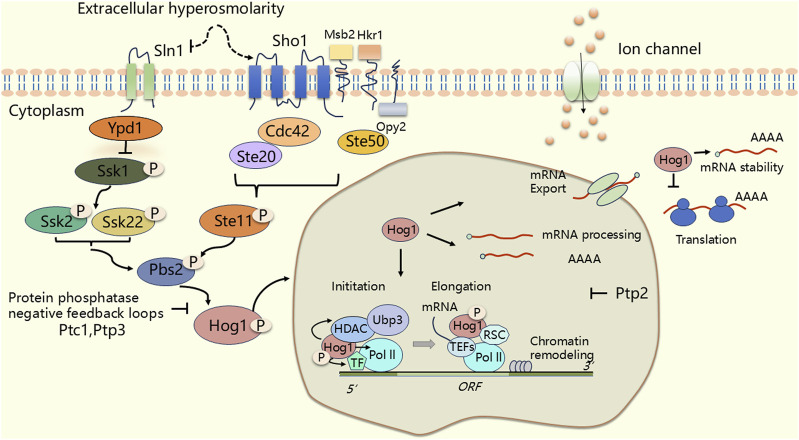
The HOG (High Osmolarity Glycerol) signal pathway in *Saccharomyces cerevisiae*. Under osmotic stress, Pbs2 integrates signals from two major independent upstream osmotic sensing pathways: the Sln1 and Sho1 branches. Upon activation, Pbs2 triggers the activation of Hog1. The activated Hog1 portion enters the nucleus and regulates transcription (RNA polymerase initiates transcription at the promoter, synthesizes the RNA chain, and continues to extend forward.). The other part of Hog1 remains in the cytoplasm and directly regulates post-translational processes such as translation. Thereby initiating a series of osmotic adaptive responses. The phosphorylation state is represented by “P”. An arrow indicates an activation state, while the T-bar symbol represents an inhibitory state.

*Saccharomyces* cerevisiae is frequently exposed to adverse environments, necessitating the evolution of regulatory mechanisms that enable stress adaptation survival (Udom et al., 2019; Yao et al., 2020; Lucena et al., 2020; Nadal and Posas, 2022). The HOG MAPK pathway in yeast, a structural and functional homologue of the mammalian p38 MAPK (Nadal and Posas, 2015), plays a crucial role in mediating cellular adaptation to stress, particularly in high osmotic pressure environments. Under osmotic stress, HOG activation occurs through two independent mechanisms, each involving a sensing mechanism and a tertiary cascade of MAPKKK (Ssk2, Ssk22, and Ste11), MAPKK (Pbs2), and MAPK (Hog1) (Duch et al., 2012). Ultimately, this cascade activates downstream substrate (Brewster et al., 1993; Maeda et al., 1994; Maeda et al., 1995; Posas and Saito, 1997; O'Rourke et al., 2002; Hohmann, 2002; O'Rourke and Herskowitz, 2004; Tatebayashi et al., 2007; Macia et al., 2009; de Nadal et al., 2011; Saito and Posas, 2012; Westfall et al., 2004) (Figure 3). One branch of this pathway involves the osmotic stress receptor Sln1, a complex variant of the well known bacterial two-component system (Maeda et al., 1994; Hohmann, 2002). In this pathway, the histidine kinase activity of Sln1 is inhibited, leading to the dephosphorylation of its downstream target, Ssk1, which activates downstream MAPKKK (Ssk2, Ssk22) (Posas et al., 1996; Posas et al., 1998; West & Stock, 2001; Horie et al., 2008) (Figure 3). The other branch features the membrane protein Sho1, which interacts with Ste20 and Ste50 to activate MAPKKK (Ste11) (Maeda et al., 1995; Posas and Saito, 1997; Drogen et al., 2000; Raitt et al., 2000; Reiser et al., 2000; Macia et al., 2009) (Figure 3). In yeast, the mucin-like transmembrane proteins Hkr1 and Msb2 are potential osmosensors and share redundant functions with Sho1 (O'Rourke and Herskowitz, 2002; de Nadal et al., 2007; Tatebayashi et al., 2007; Tanaka et al., 2014) (Figure 3). In addition, another element, Opy2, which is a transmembrane protein, acts as the membrane anchor for Ste11/Ste50 (Wu et al., 2006). The MAPKKK from both branches (Ssk2, Ssk22 and Ste11) subsequently activate the common MAPKK (Pbs2), which acts as both a scaffolding protein and a kinase, inducing the phosphorylation of Thr174 and Tyr176 to activate Hog1 (MAPK) (Brewster et al., 1993) (Figure 3). Activation of the Hog1 pathway is similar to that of the p38 MAPK pathway in mammals, and is achieved through the MAPK cascade reaction. Phosphorylated Hog1 translocates to the nucleus, where it accumulates (Ferrigno et al., 1998; Reiser et al., 1999) and recruits RNA polymerase and transcription factors [Msn2 (Schmitt and McEntee, 1996), Msn4, and Hot1 (Rep et al., 1999), Msn1 (Estruch and Carlson, 1990)] to the promoters of hyperosmolarity-associated genes [*CTT1*, *GPD1, GPD2, GPP1, GPP2*, *STL1* and *HSP12* (O'Rourke et al., 2002; Hohmann, 2002; O'Rourke and Herskowitz, 2004)], thus regulating intranuclear osmotic pressure through specific chromatin remodeling factors to ensure normal transcription and expression of relevant genes under hypertonic conditions (Posas et al., 2000; Capaldi et al., 2008; Babazadeh et al., 2014; Hohmann, 2015; Udom et al., 2019). Furthermore, *pbs2*Δ mutant or *hog1*Δ mutant, which encode MAPK kinase (MAPKK) and MAPK, respectively, lead to increased osmosensitivity and decreased glycerol levels (Brewster et al., 1993). Hog1 research reveals how transcription factors mediate gene expression in response to stress, and these mechanisms are equally applicable in mammals.

However, sustained activation of Hog1 can be detrimental to cell growth, making negative feedback regulation of HOG-MAPK signaling essential (Sacristán-Reviriego et al., 2015; Vázquez-Ibarra et al., 2020). In yeast cells, the protein phosphatases that inactivate the HOG signaling pathway are divided into two classes. The first class consists of protein tyrosine phosphatases (PTPs) specifically Ptp2 and Ptp3. Ptp2 is predominantly located in the nucleus (Mattison et al., 1999), where it binds and dephosphorylates Hog1, playing a crucial role in the negative feedback regulation of the HOG-MAPK signaling pathway (Maeda et al., 1994; Wurgler-Murphy et al., 1997; Jacoby et al., 1997; Mattison and Ota, 2000; Saito and Tatebayashi, 2004) (Figure 3). The second class comprises type 2C Ser/Thr phosphatases (PTCs) Ptc1, Ptc2 and Ptc3, which specifically dephosphorylates.

Thr174 of Hog1, thereby preventing overactivation of Hog1 phosphorylation (Warmka et al., 2001; Young et al., 2002; Sacristán-Reviriego et al., 2015). The coordinated action of activation and negative feedback regulation of the HOG signaling pathway ensures that organisms can effectively adapt to environmental changes.

The HOG pathway plays a crucial role in various cellular processes. First, it determines the short-term translation response following hyperosmotic shock, regulating protein synthesis to help the cell adapt to osmotic stress (Warringer et al., 2010; de Nadal et al., 2011). Second, the p38/HOG stress-activated protein kinase network is involved in coordinating growth and division in *Candida* albicans, although its exact role remains under debate (Sellam et al., 2019). Third, Hog1 has a dual role in the HOG pathway, acting both as a direct kinase and as a coordinator of secondary signaling mediated by effector kinases such as Rck2 (Romanov et al., 2017). In addition, under hypertonic stress, Hog1 directly binds to the n-terminal regulatory domain of Fps1, and in this case phosphorylates Rgc2 at multiple sites, shutting down the *saccharomyces* cerevisians glycerol channel Fps1, thereby regulating cellular osmotic balance (Tamás et al., 1999; Beese et al., 2009; Lee et al., 2013). Finally, in *S. cerevisiae*, Hog1 MAPK prevents crosstalk between the HOG pathway and the pheromone response MAPK pathway, ensuring specific signal transduction under osmotic stress conditions (O'Rourke and Herskowitz, 1998; Vázquez-Ibarra et al., 2020).

### HOG MAPK activated by multiple environmental stresses

In addition to its classical role under hyperosmotic stress in *S. cerevisiae*, the HOG signaling pathway has been shown to be activated by various environmental stresses. Therefore, in the section, we will primarily discuss the activation of the HOG signaling pathway in response to other environmental stressors. The Hog1 study revealed that cells adapt to environmental stress, which has similarities to the adaptation of the mammalian p38 MAPK pathway to cell physiological functions in response to environmental stress or inflammation. The aim is to provide new perspectives for the study of p38 MAPK in mammalian cells.

### Heat stress

Cells of living organisms are constantly exposed to environmental changes that can be detrimental, including rising temperatures (30°C–37°C) (Parsell and Lindquist, 1993; Piper, 1993; Dunayevich et al., 2018). These temperature increases can damage vital cell structures and impair essential biological functions (Richter et al., 2010; de Nadal et al., 2011). In response to specific stresses, cells regulate intracellular effectors and intracellular signaling pathways (de Nadal et al., 2011).

In yeast cells, there are two main classes of signaling pathways that detect and respond to sudden changes in external temperature. One pathway involves the accumulation of denatured protein 26, which is conserved in the heat-induced responses, leading to the activation of Heat Stress Factor (HSF) and the transient expression of Heat Stress Proteins (HSPs) (Franzmann et al., 2008; de Nadal et al., 2011). The other pathway responds directly to temperature changes through key heat-senstive structures, such as DNA, RNA, proteins and lipids, either by participating in or activating signal transduction pathways (Franzmann et al., 2008; de Nadal et al., 2011).

**FIGURE 4:**
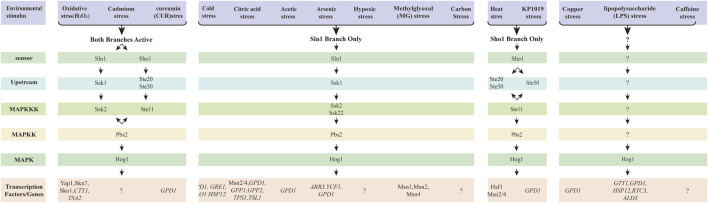
The HOG signal pathway in response to the other enviromental stresses. The HOG pathway has two branches, and different environmental stimuli can activate distinct branches, thereby regulating different transcription factors. These stresses pass through the Sln1-dependent branch, the Sho1-dependent branch, or involve both branches simultaneously. Pathway-specific protein complexes are common and necessary for signaling. Under different stress conditions, the transcription factors or genes corresponding to the bottom are different. Arrows represent the flow of information, while question marks indicate uncertainty.

Winkler A et al. demonstrated that heat stress (treated at 37°C) promotes Hog1 phosphorylation and Hog1-dependent gene expression via the Sho1 phosphorylation branch (Figure 4) (Winkler et al., 2002). Some researchers also suggest that Hog1 is involved in heat shock responses due to the transient increase in pressure (de Nadal et al., 2011). Transcription of genes such as *HSP12*, *CTT1* or *ALD3* is induced within 1–3 min of stimulation (Gasch et al., 2000; de Nadal et al., 2011). Additionally, studies have shown that the Ptp2 and Ptp3 can inactivate Hog1 to prevent excessive activation of HOG MAPK, thereby avoiding cell death (Winkler et al., 2002).

Following the activation of Hog1, the heat shock transcription factor Hsf1 and the general stress transcription factors Msn2 and Msn4 (collectively referred to as Msn2/4) are activated as master regulators of the heat shock response in *S. cerevisiae* (Figure 4) (Yamamoto et al., 2008). Hsf1 and Msn2/4 induce the expression of proteins that protect cellular components from thermal inactivation (Amorós and Estruch, 2001; Grably et al., 2002). Hsf1 is rapidly and transiently activated to counteract the detrimental effects of misfolded proteins and restore proteins homeostasis by inducing the expression of chaperones and other protective proteins under heat shock conditions (Hightower, 1990; Akerfelt et al., 2010; Rhodius and Mutalik, 2010). Additionally, Hsf1 promotes the transcription of many HSP family target genes during the recovery period following severe heat shock, including *HSP104*, *HSP82* and *HSP70* (Lindquist and Kim, 1996; Sanchez et al., 1993).

Simultaneously the transcription factors Msn2/4 were also activated to regulate the target genes such as *HSP12*, *CTT1* and *ALD3* in response to heat shock (Gasch et al., 2000; de Nadal et al., 2011). However, the sensor system and detailed mechanism of the HOG signaling pathway remain largely unexplored.

### Cold stress

Low temperatures (0°C–13°C) (Schade et al., 2004) can lead to decreased membrane fluidity (Henry and Keith, 1971; Alonso et al., 1997; Swan and Watson, 1997; Horváth et al., 1998; Hayashi and Maeda, 2006; Panadero et al., 2006), reduced enzyme activity, impaired protein translation efficiency, changes in lipid composition, disruptions in the synthesis of damaged proteins, and decreased secondary stability of DNA and RNA structures in various organisms, including yeast (Panadero et al., 2006; López-Malo et al., 2013). When yeast is exposed to low temperatures, it triggers a rapid and dynamic stress response known as the cold shock response (Aguilera et al., 2007). This response has been extensively studied in bacteria and plants, but less so in fungi. Yeast cells, in particular, HOG signal pathway has been shown to play a crucial role in adapting to cold stress, providing valuable insights into cellular mechanisms that protect against frostbite induced by low temperatures (Hayashi and Maeda, 2006; Panadero et al., 2006; Aguilera et al., 2007).

Using Western blot analysis, Hayashiet al. demonstrated that the HOG signaling pathway is activated only in a Sln1branched dependency manner when cells are exposed to cold stress (at 0 °C) (Figure 4) (Hayashi and Maeda, 2006). The transcription factors Msn2 and Msn4 bind to Stress Response Element (STRE) and are involved in the coordinated regulation of low temperature response genes (Kandror et al., 2004; Schade et al., 2004). However, there is currently no direct evidence that Hog1 induces Msn2/Msn4 expression under cold stress. Panadero et al. (Panadero et al., 2006) analyzed mRNA samples from wild-type and *hog1*Δ mutant cells using Northern blot and examined the transcription profiles of several genes. They found that the cold-induced expression of *GPD1*, *GRE1*, *GLO1* and *HSP12* was nearly completely inhibited in the *hog1*Δ mutant (Figure 4). This suggests that Hog1 may be the primary kinase regulating the expression of these genes.

Additionally, studies have shown that when cells are transferred to 12°C or 4°C, wild-type cells accumulate significant amounts of glycerol, resulting in improved freezing tolerance. In contrast, *hog1*Δ and *gpd1*Δ mutants exhibit lower survival rates after freezing. This suggests that an increased glycerol content following a temperature drop may confer frost resistance (Panadero et al., 2006). However, cells with mutations in the HOG signaling pathway grow more slowly under cold stress but are not fatal indicating that other, potentially more critical signaling pathways may also be involved in response to cold stress.

### Oxidative stress

Oxygen can cause forms of cellular damage through intermediates with differing reactivity and distribution (Bilsland et al., 2004). For instance, endogenous metabolites produced by cells, such as hydrogen peroxide (H2O2), reactive free radicals, and other oxidants, can lead to oxidative damage to proteins, lipids, and DNA (Davies, 1995; Wallace, 1998; Singh, 2000). To protect against oxidative damage, organisms constantly monitor their environment and respond to oxidative stress through signal transduction mechanisms (Lee et al., 2017). Oxidants like hydrogen peroxide, menadione, ultraviolet light and *у*-radiation can induce oxidative stress (Demple, 1998; Jamieson, 1992; Hidalgo et al., 1997; Kielbassa et al., 1997; Wallace, 1998; Zheng et al., 1998). In *S. cerevisiae*, the most typical HOG signal in the MAPK cascade is associated with oxidative stress (Singh, 2000; Haghnazari and Heyer, 2004).

Singh (Singh, 2000) found that *sln1*Δ *ssk1*Δ double mutants and *sho1*Δ *ssk2*Δ double mutants exhibited sensitivity only to H_2_O_2_ and diamine, but not to the other oxidants like menadione, ultraviolet light, and *у*-radiation. The Sho1 branch and Sln1branch were shown to protect cells only from oxidative damage caused by H_2_O_2_ and diamine, rather than other oxidants (Figure 4).

Surprisingly, a hallmark of the osmotic stress response of *S. cerevisiae* is that upstream kinases, such as MAPKK phosphorylate Hog1 kinase at tyrosine residues (Maeda et al., 1994; Schüller et al., 1994; Gustin et al., 1998). However, when yeast cells were exposed to H_2_O_2_, Hog 1 tyrosine phosphorylation did not increase. Similarly, no significant increase in tyrosine phosphorylation was observed after exposure to other oxidants, consistent with the findings of Schüller et al. (1994). These results suggest that direct phosphorylation of Hog1 is not involved in H_2_O_2_- induced stress signaling. Interestingly, the hog1Δ mutant was sensitive to H_2_O_2_, while the hog1Δ mutant expressing mouse p38 kinase demonstrated resistance to H_2_O_2_. Thus, the expression of p38 kinase compensates for the loss of Hog1 and protects cells from H_2_O_2_-induced damage (Singh, 2000).

Lee et al. (2017) found that Hog1 MAPK could be activated by oxidative stress (specifically H_2_O_2_) through spot dilution assay and Western blot analysis. However, the activation mechanism was shown to only through Ssk1-Ssk2 in Sln1 branch, rather than Ssk1-Ssk22. While only a few cells exhibited nucleus localization of Hog1 upon H_2_O_2_ exposure, most were remained in the cytoplasm. Additionally, Lee YM et al. detected that both Hog1 and Rck2 were activated and phosphorylated upon H_2_O_2_ stress. Interestingly, the number of cells with Hog1 in the nucleus was significantly higher in *rck*Δ mutant cells compared to wild-type cells (Bilsland et al., 2004).

Several important transcription factors, including Yap1, Skn7 and Sko1, which are regulated by Hog1, play crucial roles in the oxidative stress response. These factors are necessary for the regulation of genes involved in protection against oxidative damage (Figure 4) (Rep et al., 2001). Furthermore, the genes of *CTT1* and *TSA2*, which encode antioxidants, are induced in respond to oxidative stress by these transcriptions factors (Schüller et al., 1994; Wong et al., 2003). Massive genes associated with the CRE and STRE elements, regulated by Hog1, are induced under oxidative conditions (Lee et al., 2017; Yaakoub et al., 2022). Currently, it remains controversy regarding the signaling pathway involved in the oxidative stress response. Much work and further investigation are needed in this research area to address existing challenges.

### Acid stress

Acids can be categorized as weak or strong, and as organic or inorganic. Regardless of the type, the growth of organisms requires adaptation to external hydrogen ion concentrations (Lawrence et al., 2004). Acid stress is influenced not only by the toxicity of high concentrations of hydrogen ions but also depends on the chemical nature of the acid to which the organism is exposed (Lawrence et al., 2004). In fact, different properties of acids can produce different inhibitory effects; for instance, different weak organic acids can exert significantly different inhibitory effects on microorganisms, even at the same pH (Salmond et al., 1984). In *S. cerevisiae*, the HOG MAPK pathway has been showed to regulate resistance to citric acid as well as acetic acid during acid stress (Lawrence et al., 2004; Mollapour and Piper, 2006).

Citric acid, an intermediate metabolite of the tricarboxylic acid (TCA) cycle, is a key component of normal respiratory metabolism in *S. cerevisiae* and is commonly used as a preservative to prevent microbial growth. Lawrence et al. (Lawrence et al., 2004) were the first to discover that the HOG MAPK pathway is vital for the regulation of citric acid stress adaptation, utilizing screening *S. cerevisiae* cleavage, transcriptional analysis, and determination of protein expression changes. By screening the *S. cerevisiae* mutant cells, including *HOG1*, *SSK1*, *MSN2*, *PBS2*, *PTC2*, *PTP2* and *PTP3*, it was confirmed that the HOG MAPK pathway was activated by the Sln1 branch upon citric acid stress (Figure 4). The genes regulated by the HOG pathway, which are involved in glycerol and trehalose metabolism (e.g., *GPD1*, *GPP1*, *GPP2*, *TPS1*, and *TSL1*) (Figure 4), general stress response (e.g., *CTT1*, *HSP42*, and *DDR48*), and cell wall integrity (e.g., *SPI1* and *CWP1*), were upregulated in wild type yeast cells under citric acid stress. Additionally, genes such as *CTT1*, *ALD3*, *PNC1*, *DDR48* and *YDL204w*, which serve as marker for the transcription factor Msn2/4, were also upregulated, indicating the activation of Msn2/4 in response to citric acid stress. Moreover, Hog1 was found to negatively regulate the expression of Bmh1p, Pdb1p, Ura1p, Fba1, Ydr533cp, Gnd1p and Car1p during citric acid exposure. The study clearly demonstrated that the HOG MAPK pathway was activated by the Sln1 branch to regulate gene expression to alter biological processes and cause glycerol accumulation in response to citric acid stress (Lawrence et al., 2004). Furthermore, the HOG MAPK pathway was similarly activated by the Sln1 branch in response to acetate stress (Mollapour and Piper, 2006). The *GPD1* gene and glycerol content were induced only in an acetate culture with a pH of 6.8, but not in culture with a pH of 4.5 (Mollapour and Piper, 2006; Ludovico et al., 2001; Ludovico et al., 2003). In summary, the HOG pathway by Sln1 branch is activated in response to both citric acid and acetic acid stress. However, the mechanism governing gene expression following the activation of the HOG signaling pathway remains unclear.

### Heavy metal

The widespread use of heavy metals and their improper disposal pose a serious threat to the environment and human health. While transition metals, heavy metals and quasi-metals can be toxic, some transition metals are essential trace elements necessary for biological function. All cells have mechanisms for metal ion homeostasis. typically involving a balance between uptake and efflux systems (Tamás and Wysocki, 2001; Rosen, 2002). Heavy metals are cofactors for several microbial enzymes and are present at low concentrations required for normal biological function in yeast (Kirchman and Botta, 2007). However, when the concentration exceeds permissible thresholds, heavy metals can impair cellular function and viability (Dong et al., 2013; Jiang et al., 2014; Gerwien et al., 2018; Ren et al., 2022).

In *S. cerevisiae*, regulatory mechanisms for developing tolerance to various metals have been identified, including heavy metals such as copper (Ren et al., 2022), cadmium (Jiang et al., 2014; Zhao et al., 2021), iron (Martins et al., 2018)), as well as metalloids like arsenic (Sotelo and Rodríguez-Gabriel, 2006; García et al., 2004; Lee and Levin, 2018), antimony (Thorsen et al., 2006). These mechanisms are crucial for the survival and adaptation of yeast in environments contaminated with toxic metals.

### Copper

Copper is essential for life, yet it can become toxic when its concentration exceeds physiological limits. In cellular contexts, copper ions can exist in various valence states (Ren et al., 2022). The toxicity of copper primarily arises from its REDOX properties, enabling it to participate in Fenton-like reactions that generate harmful reactive oxygen species (ROS) (Ren et al., 2022), This leads to cellular damage, including protein oxidation, DNA and RNA cleavage, and lipid peroxidation that compromises membrane integrity (Dong et al., 2013; Gerwien et al., 2018; Castro et al., 2021).

Ren M et al. demonstrated for the first time that copper exposure induce oxidative stress, including increased levels of ROS and malondialdehyde (MDA), the enzymes activity of GSH and SOD, and upregulated expression of related genes to protect cells defend against oxidative toxicity (Ren et al., 2022). In addition, trace amounts of Hog1 were activated under copper stress, leading to the upregulation of gene expression for *CTT1*, *GPD1*, *HSP12*, *RTC3*, and *ALD3*, as confirmed through phenotypic assays, Western blot assays and RT-PCR (Ren et al., 2022) (Figure 4). However, the specific branch regulating Hog1 activation in response to copper remains unidentified. Furthermore, copper exposure resulted in significant cell cycle arrest in G1 phase, while Hog1 was partially involved in regulating cell cycle progression (Ren et al., 2022). Despite these findings, the HOG pathway response to copper exposure is under-researched, and the precise mechanisms involved are still unclear.

### Cadmium

Cadmium is a highly toxic trace elements that accumulates in organisms, (Genchi et al., 2020; Farooq et al., 2020; El-Esawi et al., 2020; Ozturk et al., 2021), triggering a cascade of death and adaptive signals in eukaryotic cells, including the formation of oxidants as well as the prevention of DNA damage and DNA repair (Zhang and Reynolds, 2019). It is classified as a carcinogen affecting various tissues (Bertin and Averbeck, 2006; Bishak et al., 2015; Larsson et al., 2015). The HOG pathway identified as crucial for yeast cells in resisting cadmium-induced toxicity (Ozturk et al., 2021; Zhao et al., 2021).

In Jiang et al. employed phenotypic screening of mutants lacking components of the HOG signaling pathway alongside Western blot analysis, revealing that the upstream branches Sln1 and Sho1 can activate the HOG signaling pathway in response to cadmium stress (Figure 4) (Jiang et al., 2014). Notably, the MAPKKK involved in cadmium signaling within the Sln1 branch was identified as Ssk2, rather than Ssk22 (Jiang et al., 2014) (Figure 4). *CYS3*, *CYS4*, and *GSH1*, which are involved in cysteine and glutathione biosynthesis, were identified by transcriptomic, proteomic, and degenomic methods, suggesting that cysteine and glutathione are essential for cadmium tolerance in yeast cells (Jiang et al., 2014). It was observed that ROS levels and cell death levels were induced under the influence of Cd in mutant cells such as *hog1*∆ mutant and *pbs2*∆ mutant (Zhao et al., 2021). Furthermore, cadmium-induced Hog1 phosphorylation was shown to require the Unfolded protein-response (UPR) pathway (Zhao et al., 2021). The loss of *HAC1* and *IRE1* was found to enhance the nuclear accumulation of Hog1 and increase *Slt2* localization in the cytoplasm and bud neck, suggesting that both Hog1 and *Slt2* are crucial for regulating cellular processes in the absence of the UPR signaling pathway (Zhao et al., 2021). However, the mechanisms by which the HOG signaling pathway regulates downstream transcription factors and gene expression remain unclear.

### Arsenic

Arsenic is a toxic metalloid that is widely present in the environment, and exposure has been linked to various diseases, including liver, kidney, and lung cancers (Evens et al., 2004; Thorsen et al., 2006). Despite their toxicity, arsenic-containing drugs have become part of modern therapies (Thorsen et al., 2006). Studies involving arsenite have shown that activation of the HOG pathway is crucial for tolerance in *S. cerevisiae*.

Sotelo and Rodríguez-Gabriel (2006) first demonstrated that Hog1 is rapidly phosphorylated in response to arsenic stress and triggers a transcriptional response via the Sln1 branch of the HOG pathway (Figure 4). The abundance of several mRNAs in response to sodium arsenic in the wild type and the *hog1*Δ mutant was examined by quantitative reverse transcript PCR (García et al., 2004). Among the monitored mRNAs, the transcription factor Arr1, which is critical for upregulating several genes involved in the sodium arsenic response (Bouganim et al., 2001; Menezes et al., 2004), including the plasma membrane transporter *ARR3*; the vacuolar transporter *YCF1*; and Glycerol-3-phosphate dehydrogenase *GPD1*, which plays a crucial role in hypertonic stress responses and is regulated by Hog1 activity (Albertyn et al., 1994), it was observed that *ARR3* mRNA was strongly induced under arsenic treatment, whereas its induction was significantly diminished in *hog1*Δ mutant cells (Sotelo and Rodríguez-Gabriel, 2006) (Figure 4). The defective induction of *ARR3* and *YCF1* expression is consistent with the high sensitivity of the *hog1*Δ mutant to sodium arsenic (Sotelo and Rodríguez-Gabriel, 2006). Furthermore, analysis of strains lacking the transcription factors Sko1, Msn2/4, Hot1, and Smp1, all of which are regulated by Hog1, it was found that Hog1 regulated the arsenic response independently of these factors (Sotelo and Rodríguez-Gabriel, 2006). Thorsen M et al. confirmed the above results and showed that the increase in arsenite influx was dependent on the aqueous triglyceride protein Fps1 (Thorsen et al., 2006).

Lee and Levin (2018) investigated the two main forms of inorganic arsenic: trivalent arsenate [As (III)] and pentavalent arsenate [As (V)]. Their findings revealed that Hog1 is activated only when As (III) is converted to Mas (III), a metabolite that activates Hog1 by inhibiting its tyrosine-specific phosphatases Ptp2 and Ptp3. These phosphatases typically maintain Hog1 in a state of low activity, representing a negative feedback mechanism within the HOG pathway. Hog1 is activated through arsenate [As (V)] through a MAPK cascade (Lee and Levin, 2018; Lee et al., 2019), a mechanism that differs from the activation by As (III). Both As (III) and As (V) stimulate the expression of arsenic-protected genes, including *ACR2* and *ACR3* genes, through the ap-1 like transcription factor ACR1 (Wysocki et al., 2004). Notably, the induction of *ACR3* by As (III) was found to be partially dependent on Hog1, while its induction by As (V) occurred independently of Hog1. Despite these insights, the underlying mechanisms remain unclear.

### Lipopolysaccharide (LPS)

A typical activator of the immune and inflammatory systems is lipopolysaccharide (LPS), a component of the outer leaflets of the cell wall of Gram-negative bacteria, commonly referred to as bacterial endotoxin. LPS triggers systemic changes associated with infectious shock (Han et al., 1994; Marques et al., 2006).

Since p38 mediates the action of LPS in mammalian cells, Marques et al. (Marques et al., 2006) investigated the adaptive response of the HOG pathway to LPS in *S. cerevisiae.* They found that exposure to LPS induced Hog1 phosphorylation and translocation to the nucleus, where it is in a catalytically active state and upregulates *GPD1* mRNA levels (Figure 4). However, further exploration is needed to elucidate the upstream branch of the HOG signaling pathway and their regulatory effects on gene expression.

### Curcumin (CUR)

CUR, an active polyphenol derived from the spice turmeric, has garnered significant attention for its diverse therapeutic and preventative applications. CUR has been used as a dietary supplement for many years and is widely used in Ayurvedic medicines (Aggarwal et al., 2007). CUR has good therapeutic potential and may play an important role in the prevention of neurodegenerative disorders, many forms of cancer including colon and pancreatic cancer cancers, intestinal disorders, and other disorders (Gupta et al., 2013; Shehzad et al., 2013; Monroy et al., 2013; Vera-Ramirez et al., 2013). However, the exact mechanism underlying these effects remain under investigation (Azad et al., 2014). New aspects of the CUR-induced stress response in *S. cerevisiae* have provided additional understanding of the treatment of CUR. Azad G. K et al. first discovered that CUR exposure causes Hog1 phosphorylation in *S. cerevisiae* across both Sln1 and Sho1 branches and the Ssk2 in the Sln1 branch is required for CUR-induced Hog1 to achieve optimal phosphorylation by Western blotting and mutant cell assays to achieve (Figure 4) (Azad et al., 2014). The expression of *GPD1* regulated by Hog1 was significantly increased by RT-PCR. Meanwhile, immunoblotting indicated a notable reduction in phosphorylated Hog1 levels following the addition of iron, suggesting that CUR-induced iron deficiency contributes to Hog1 phosphorylation, which can be restored by iron supplementation (Azad et al., 2014). However, the specific transcription factors and gene expressions regulated by Hog1 remain clear.

### Hypoxic stress

Oxygen is a critical electron acceptor in aerobic respiration and is essential for the biosynthesis of important cellular components such as steroids, unsaturated fatty acids (UFAs), and hemoglobin (Rosenfeld and Beauvoit, 2003; Hickman et al., 2011). However, oxygen also has a downside side, during metabolism, it produces reactive oxygen species, which can damage cellular components (Jamieson, 1998; Hickman et al., 2011). To adapt to changes in oxygen levels in the environment, most organisms respond to changes in oxygen levels through different mechanisms. In *S. cerevisiae*, the response to hypoxia is regulated via the HOG signaling pathway.

Hickman et al. studied hypoxia induction in *S. cerevisiae* by analyzing the hypoxic-induced seripauperin (PAU) gene and showed for the first time that Hog1 is phosphorylated and involved in the hypoxic growth response by phosphorylation-specific antiserum analysis and Western blot analysis (Hickman et al., 2011). Additionally, the *ssk1*Δ *ste11*Δ double mutants exhibited the same defect as the *ssk1*Δ single mutant, suggesting that Ste11 does no play a role in the hypoxic activation of Hog1. This indicates that the upstream Sln1 branch of the HOG pathway is involved in hypoxia stress (Figure 4). Under hypoxia conditions, the mRNA levels of three hypoxia genes (*DAN1*, *INO1* and *TDH1*) were also increased in the *hog1*Δ mutant. This suggests that HOG signaling pathway is not the primary signaling pathway for hypoxia stress, and other pathways may play a more central role in this adaptive response.

### Caffeine

Caffeine, a purine analogue of methylxanthines, occurs naturally in many plants as a secondary metabolite. In plants, caffeine serves a protective role and acts as an insecticide, paralyzing and killing herbivorous insects (Elhasi and Blomberg, 2023). While caffeine has both positive and negative effects on human health, its ability to modulate various neurotransmitter systems can significantly affect physiological functions. *S. cerevisiae* is highly responsive to caffeine, which influences cell growth, morphology, DNA repair, intracellular calcium homeostasis, and cell cycle progression (Kuranda et al., 2006; Elhasi and Blomberg, 2023). Elhasi and Blomberg found that caffeine treatment induces rapid, intense, and transient phosphorylation of Hog1 (Elhasi and Blomberg, 2023). Phosphorylated Hog1 is immediately accumulated in the nucleus under caffeine stress with HOG1-GFP cells (Figure 4) (Elhasi and Blomberg, 2023). However, the specific pathway through which caffeine affects Hog1 phosphorylation remains unclear, and the mechanism of cellular transcription following activation require further investigation.

### Methylglyoxal (MG)

MG is a glycolytic by product formed during the dephosphorylation of dihydroxyacetone phosphate (DHAP), which is an intermediate in the interconversion of DHAP and glyceraldehyde phosphate (GA3P) (Phillips and Thornalley, 1993). The production of MG in *S. cerevisiae* occurs as a non-enzymatic, spontaneous process (Martins et al., 2001). In addition, MG is known to have significant toxic effects, influencing DNA and proteins (Vander Jagt et al., 1992). To prevent the overaccumulation of MG, yeast cells initiate a genetic response when internal concentrations reach a certain threshold.

Aguilera J et al. showed that the HOG signaling pathway regulates the genetic response of yeast to MG (Aguilera et al., 2005). Western blot analyses revealed that *hog1*Δ mutant and upstream *sln1*Δ branch mutant exhibited sensitivity and impairment under MG treatment, while *sho1*Δ *ssk1*Δ double mutants displayed an attenuated response similar to that of *ssk1*Δ single mutant. This indicates that MG response primarily depends on the Sln1 branch (Figure 4). The absence of Hog1 protein led to reduced MG-dependent mRNA accumulation of the three reporter genes *GPD1*, *GLO1* and *GRE3* (Aguilera et al., 2005). Furthermore, the combined deletion of the transcription factors Msn1, Msn2 and Msn4 virtually eliminated the accumulation of *GLO1*, *GRE3*, and *GPD1* mRNAs (Figure 4) (Aguilera et al., 2005).

Interestingly, MG does not induce hyperphosphorylation of Hog1 or its nuclear translocation in the parental strains (Aguilera et al., 2005). Although the phosphorylated form of Hog1 is crucial for transcriptional activity, dual phosphorylation of Hog1 is essential for triggering transcriptional responses (Aguilera et al., 2005). The activity of the HOG pathway enhance the expression of MG response genes under both non-induced and inducible conditions, thereby protecting cells from this toxic glycolytic byproduct (Aguilera et al., 2005). However, studies on MG stress are limited, the specific mechanism involved remain unclear.

### KP1019

Ruthenium is a non-essential transition metal known for its diverse coordination chemistry, making it an attractive candidate for development of pharmacologically active compounds. Among these, KP1019 has emerged as a promising ruthenium-containing drug candidates for cancer therapy. Research indicates that KP1019 induces DNA damage in *S. cerevisiae*, resulting in delayed cell cycle progression and ultimately leading to cell death (Stevens et al., 2013). Singh et al. were the first to investigate the tolerance of the HOG pathway to KP1019. Their phenotype and Western blotting analyses demonstrated that KP1019 induces the Hog1 phosphorylation (Singh et al., 2014). Additionally, it was found that *GPD1* mRNA level was upregulated within 30 min after KP1019 treatment by RT-PCR (Singh et al., 2014). Experiments involving mutant deletion strains revealed that Hog1 activation is primarily regulated by the Sho1 branch (Figure 4) (Singh et al., 2014). Therefore, the activation of HOG pathway by KP1019 is mediated by the Sho1 branch. Despite the potential of KP1019 as a novel anticancer agent, little is known about the specific biological pathways and molecules it targets (Singh et al., 2014).

### Carbon stress

Yeast cells detect external nutrient levels through signaling pathways that regulate metabolism and transcription. They preferentially utilize glucose and fructose as carbon sources and favors fermentation over oxidative phosphorylation to harness energy and precursor molecules for biosynthesis. Consequently, yeast cells can rapidly and extensively modify their transcriptional programs in response to fluctuations in glucose levels (Vallejo and Mayinger, 2015). Piao H et al. reported that glucose starvation induced Hog1 phosphorylation (Piao et al., 2012). During glucose starvation, Hog1 phosphorylation is slower and completely dependent on Ssk1,but not on Sho1 (Vallejo and Mayinger, 2015). However, the specific transcription factors involved and the resulting changes in mRNA levels remain unclear.

## Conclusion

From single cells to mammals, organisms respond to a variety of environmental stimuli through the MAPK signaling pathway to maximize survival. In mammals, the p38 signaling pathway is a crucial MAPK signaling pathway that significantly impacts human health and serves as an important area of research for various diseases. p38 plays a role not only in cellular regulation but also as a therapeutic target for conditions such as immune response, neurodegenerative diseases, inflammation, cancer, and viral infections. Although p38-related drugs have been extensively studied in clinic settings, their successes have been limited, and practical applications remain scarce. Thus, accurate and in-depth studies on p38 are still ongoing.

The main role of Hog1 is to regulate the response of cells to changes in the external environment under stress conditions such as high osmotic pressure. In mammals, the p38 MAPK pathway has a similar structure and function, especially in cellular stress response. Therefore, the study of Hog1 activation mechanism provides a framework for understanding the activation of p38 in mammals.

In the Hog1 signaling pathway, several key proteins mediate signaling processes, including kinases, splices, and transcription factors. For example, Ypd1 and Ssk1 regulate Hog1 activation upstream. Similarly, in mammals, p38 activates downstream signaling through the regulation of its upstream kinases, such as MKK3/MKK6. The Hog1 study provides a better understanding of how these transcription factors respond to environmental stress, as well as how p38 regulates stress responses through similar signaling mechanisms, and advances research into the role of these factors in mammalian systems. p38 can activate or inhibit the Hog1 signaling pathway through certain conditions, and *vice versa*. An understanding of this interplay can help develop more nuanced intervention strategies. The mammalian p38 MAPK pathway and the yeast HOG pathway are highly homologous in structure and function. *S. cerevisiae* as a well-studied model eukaryote, provides insights into the conserved of the HOG pathway across eukaryotes. We hypothesize that by examining the changes in *S. cerevisiae* in response to environmental stimuli, in conjunction with studies on the p38 signaling pathway, we can gain valuable insight into the role of p38 in human disease and health. Research on the stress response of *S. cerevisiae* can enhance our understanding of stress response in microorganisms and mammals, ultimately aiding the development of therapeutic strategies for various diseases and contributing to improved human health. Future studies should focus on the HOG pathway of resistance mechanisms under various adverse conditions, refining existing models. Future studies can start from the aspects of targeting specific transcription factors, kinase interactions, signaling pathway feedback mechanisms, etc., to provide new targets and strategies for the treatment of various diseases (such as cancer, inflammatory diseases, neurodegenerative diseases, etc.). Cross-species comparative studies, especially the analogy between Hog1 and p38, will provide theoretical basis and practical guidance for the development of more accurate and effective targeted therapies. and exploring new access mechanisms. Further exploration of the relationship between the HOG and p38 MAPK pathways in response to stress is necessary, including identifying specific transcription factors or promoters involved. Another layer of the relationship between HOG pathway and p38 MAPK pathway in response to adversity, whether there are homologues, is not clear, whether kinases such as Hog1 and their dependent factors can help p38 in disease management and treatment. Given the significance of the Hog1/p38 signaling pathway in both yeast and mammalian cells, further investment in understanding this mechanism is warranted for the advancement of human health.
